# Polarization‐resolved femtosecond Vis/IR spectroscopy tailored for resolving weak signals in biological samples using minimal sample volume

**DOI:** 10.1002/2211-5463.70180

**Published:** 2025-12-23

**Authors:** Clark Zahn, Karsten Heyne

**Affiliations:** ^1^ Department of Physics Free University Berlin Germany

**Keywords:** anisotropy, continuum band, infrared spectroscopy, polarization resolved spectroscopy, ultrafast protein dynamics, ultrafast spectrocsopy

## Abstract

Photoreceptor proteins have a variety of functions and properties that are utilized in cells. The starting point is the absorption of light by a chromophore, triggering a cascade of specific reactions in the protein lasting from femtoseconds to seconds. To understand the individual early reaction steps, we need to trace the ultrafast structural dynamics. In this report, we present our approach towards improving the signal‐to‐noise ratio in polarization‐resolved transient infrared (IR) spectroscopy, enabling such measurements. We outline our polarization‐resolved ultrafast visible (Vis)‐pump/IR‐probe spectroscopy setup using IR referencing. The setup is tailored towards tracing small signals of photo‐degrading biological samples (e.g. site‐specific mutant proteins, that are available only in limited quantities). We provide a comprehensive overview on characterizing the excitation conditions for polarization resolved measurements. The obtained spectra allow for direct tracing of reaction dynamics and conformational changes of molecular groups within the chromophore, protein side chains and hydrogen‐bond network.

## Graphical abstract

Unique biological samples, such as site‐specific mutant proteins, are available only in limited quantities. Here, we present a polarization‐resolved transient infrared spectroscopy setup with referencing to improve signal‐to‐noise tailored towards tracing small signals. We provide an overview of characterizing the excitation conditions for polarization‐resolved measurements. The obtained spectra enable direct tracing of reaction dynamics and conformational changes within the chromophore, protein, and hydrogen‐bond network.

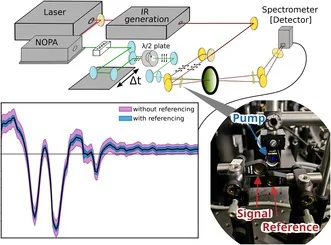

**Abbreviations**
AOPDF: acousto‐optic programmable dispersive filter
CB: continuum band
IR: infrared
NOPA: non‐collinear optical parametric amplifier
S/N: signal‐to‐noise ratio
tdm: transition dipole moments
Vis: visible

Many biological processes, such as vision, photosynthesis and enzymatic light sensing, are initiated by the absorption of visible light, triggering a cascade of processes on timescales ranging from femtoseconds to milliseconds. Capturing the early events of the photoreaction is essential for understanding the functional mechanisms of light‐sensitive proteins and cofactors. Here, polarization‐resolved femtosecond visible (Vis)‐pump/infrared (IR)‐probe spectroscopy is a powerful technique for investigating the ultrafast structural dynamics in photoactive biological systems. On the one hand, vibrational modes are sensitive to structural rearrangements in the local chemical structure, hydrogen bonding and conformation. On the other hand, measuring the anisotropy provides information on the relative orientation between the initial electronic excitation and the probed vibrational transition dipole moments (tdm) [1, 2]. This allows us to disentangle spectrally overlapping features and identify vibrational bands based on their orientation or reveal heterogeneity in the absorption spectrum [3].

## Materials

Depending on the specifications of the laser system and experimental requirements, a broad range of experimental configurations are possible, ranging from commercial systems to customized, home‐built setups. The specific choice of setup depends on the experimental requirements, such as the desired spectral coverage and temporal resolution. Generally speaking, for the pump pulse, it is advantageous to be easily spectrally tunable across the visible spectrum and also temporally compressible. This allows selective excitation of specific chromophores and electronic transitions for different samples. The probe pulse should offer a broad spectral coverage in the mid‐infrared region, allowing to trace changes for different vibrational bands specific to the investigated sample.

In our current setup (Fig. 1), a Nd:YAG laser system (Pharos; Light Conversion, Vilnius, Lithuania) delivers 330 fs pulses at 1030 nm, operated at 1 kHz with a power of 3 W. Mid‐IR probe pulses are generated by a commercially available broad‐bandwidth mid‐IR Optical Parametric Amplifier (Orpheus and Lyra; Light Conversion), providing readily tunable (50–100 fs) pulses in the spectral range from 2500–10 000 nm (1000–4000 cm^−1^) with a bandwidth of 200–300 cm^−1^. IR pulses are detected by dispersing the beam with an imaging spectrograph and recorded by a 128 × 128 element MCT‐array (2DMCT; PhaseTech, Madison, WI, USA) with a spectral resolution of 1–4 cm^−1^. Spectrally tunable pump pulses are generated by a home built two‐stage/three stage non‐collinear optical parametric amplifier (NOPA) [4], generating output pulses of 10–20 μJ. An acousto‐optic programmable dispersive filter (AOPDF) (Dazzler; Fastlite, Bordeaux, France) is used for further pulse manipulation. The AOPDF pulse shaper is used for compression of the pump pulse to 50–100 fs and spectral filtering, allowing for selective excitation at specific wavelength. A λ/2‐plate is used to set the polarization of the pump‐beam, alternating between perpendicular and parallel pump–probe configuration.

**Fig. 1:**
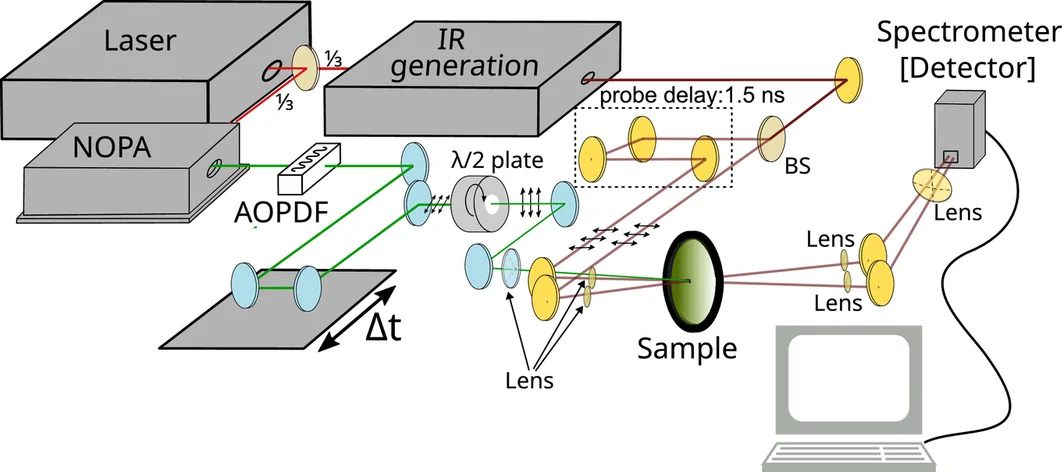
Schematic of the visible‐pump/infrared‐probe spectroscopy setup with referencing. After IR generation, the probe beam is split into a signal and reference beam. The signal is delayed by 1.5 ns by extending the path length. After passing the sample, both signal and referencing are dispersed by a spectrometer and simultaneously detected. Tunable pump pulses are generated by a home built NOPA and passed through a AOPDF for further pulse manipulation. After delaying the pump pulse with a mechanical delay stage, a rotational λ/2‐plate changes the polarization of the pump‐beam before the pump pulse is directed into the sample. BS, beamsplitter 70%/30% (signal/ref).

## Methods

### Sample preparation

Biological samples, particularly site‐specific mutant proteins, are often available only in limited quantities, making experimental setups requiring large sample volumes impractical. Moreover, the high viscosity of highly concentrated protein solutions further constrains sample preparation. To address these challenges and minimize sample consumption, samples are prepared in custom‐built sample cells with diameters of 1 or 2 inches, where the sample is sealed between two barium fluoride windows using a 50‐ or 100‐μm teflon spacer (Fig. 2). This allows to use very small amounts of sample volume of < 50 μL. However, for biological samples with a long photocycle and cumulative photo‐degradation, the sample must be moved during measurement. This introduces additional noise from sample inhomogeneity and slight beam deflections caused by small variations in the cell windows and spacers.

**Fig. 2:**
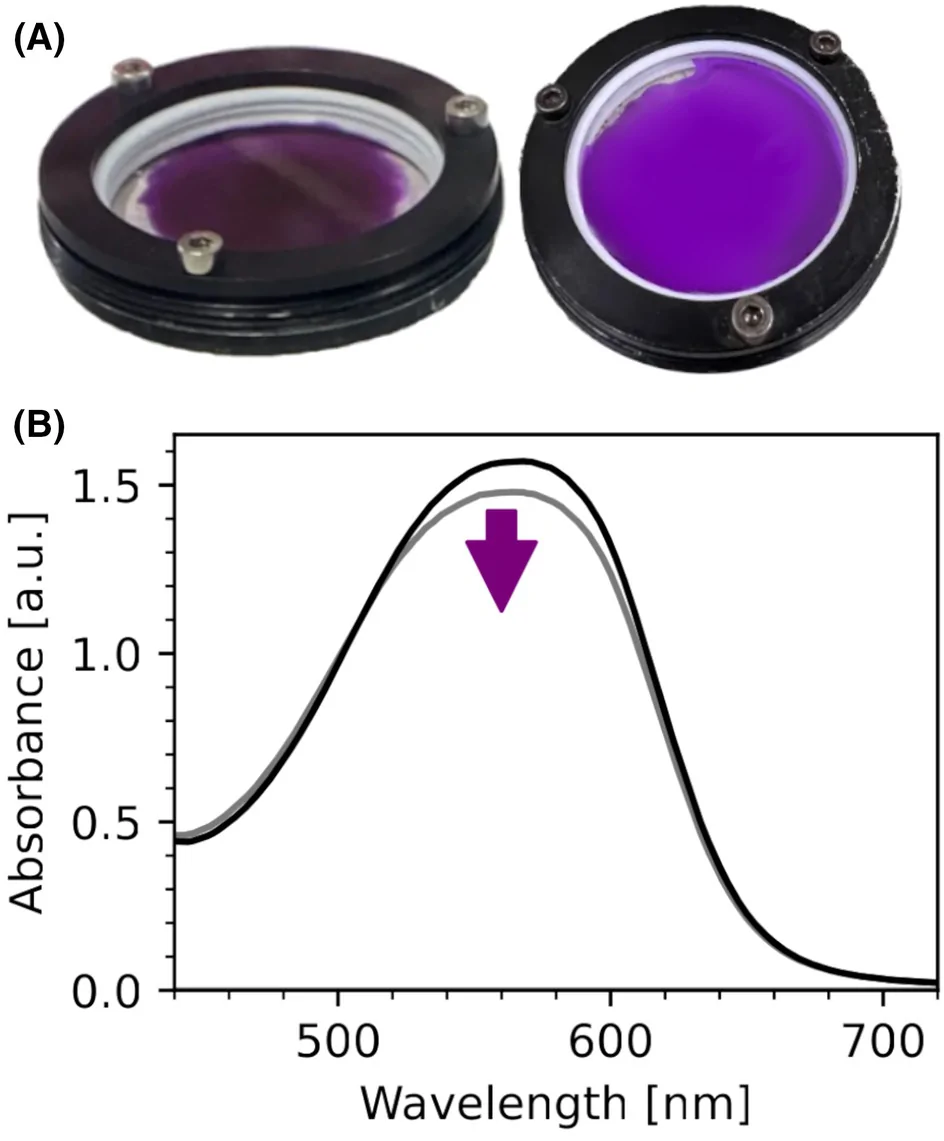
(A) Bacteriorhodopsin prepared in a 2‐inch sample cell with 100‐μm spacer. (B) Decrease (black → gray) in visible absorption of Bacteriorhodopsin as a result of accumulative photo‐degradation after approximately 50 000 shots at 0.4 ± 0.1 mJ/180 ± 50 μm.

### Sample movement/sample degradation

The sample is moved with a Lissajous‐scanner consisting of a home built sample holder mounted on two translation stages (PIMag V‐408 and M‐414; both Physik Instrumente, Karlsruhe, Germany), ensuring a fresh sample volume between consecutive pump pulses. Depending on the movement settings, it takes on average approximately 30 s to return to the same position. Yet, cumulative photo‐degradation leads to a decrease in visible absorption (Fig. 2). However, photo‐degradation is highly sample specific and cannot be generalized. For Bacteriorhodopsin, Kovacs et al. [5] performed measurements at different excitation energies. They recorded 70 scans with 600 shots per data point at 0.3 mJ/200 μm, with no observable sample change. At 0.7 mJ/200 μm, they used two samples, measuring 30 scans with 600 shots per sample, showing moderate degradation over time. At 1.4 mJ/200 μm they used six samples measuring eight scans with 600 shots, showing strong degradation.

### IR referencing for signal/noise reduction

Referencing schemes are well established in heterodyned spectroscopy, with modern implementations reaching the noise floor of the detector [6, 7, 8]. Although this suppresses noise from intensity fluctuations, it does not suppress noise from sample inhomogeneity and variations in the sample cell. Thus, to improve the signal‐to‐noise ratio (S/N), even when the sample is moved, we implemented a refined referencing scheme (Fig. 3). We divide the IR probe beam into two beams designated as signal and reference, with pulse energies of 100 ± 50 nJ/200 μm. Both beams are directed to pass through the exact same spatial location on the sample, with the reference beam being temporally offset by increasing the path‐length of the signal. This ensures that the reference beam arrives few nanoseconds (approximately 1.5 ns) before the signal, serving as a background measurement. Unfortunately, because the reference beam has to pass the exact same spatial location, spatial constraints do not allow for simultaneous detection of parallel and perpendicular pump/probe polarizations, which would further mitigate sample degradation and shorten measurement time. After passing the sample, both signal $I_{\text{signal}}\left(\nu \right)$ and reference $I_{\mathrm{ref}}\left(\nu \right)$ are simultaneously detected on the detector. The measured signal *S* is computed by normalizing $I_{\text{signal}}$ with $I_{\mathrm{ref}}$, as:

$$\begin{array}{l} \;I_{\text{probe}}\left(\nu \right)=\frac{I_{\text{signal}}\left(\nu \right)}{I_{\mathrm{ref}}\left(\nu \right)}\\ \Rightarrow S\left(\nu \right)=-\log\left(\frac{\sum_{i=0,2\ldots }I_{\text{probe}}\left(i,\nu \right)}{\sum_{j=1,3\ldots }I_{\text{probe}}\left(j,\nu \right)}\right) \end{array}$$

where the indices *i* and *j* represent the *i*‐th pumped and *j*‐th unpumped shot.

**Fig. 3:**
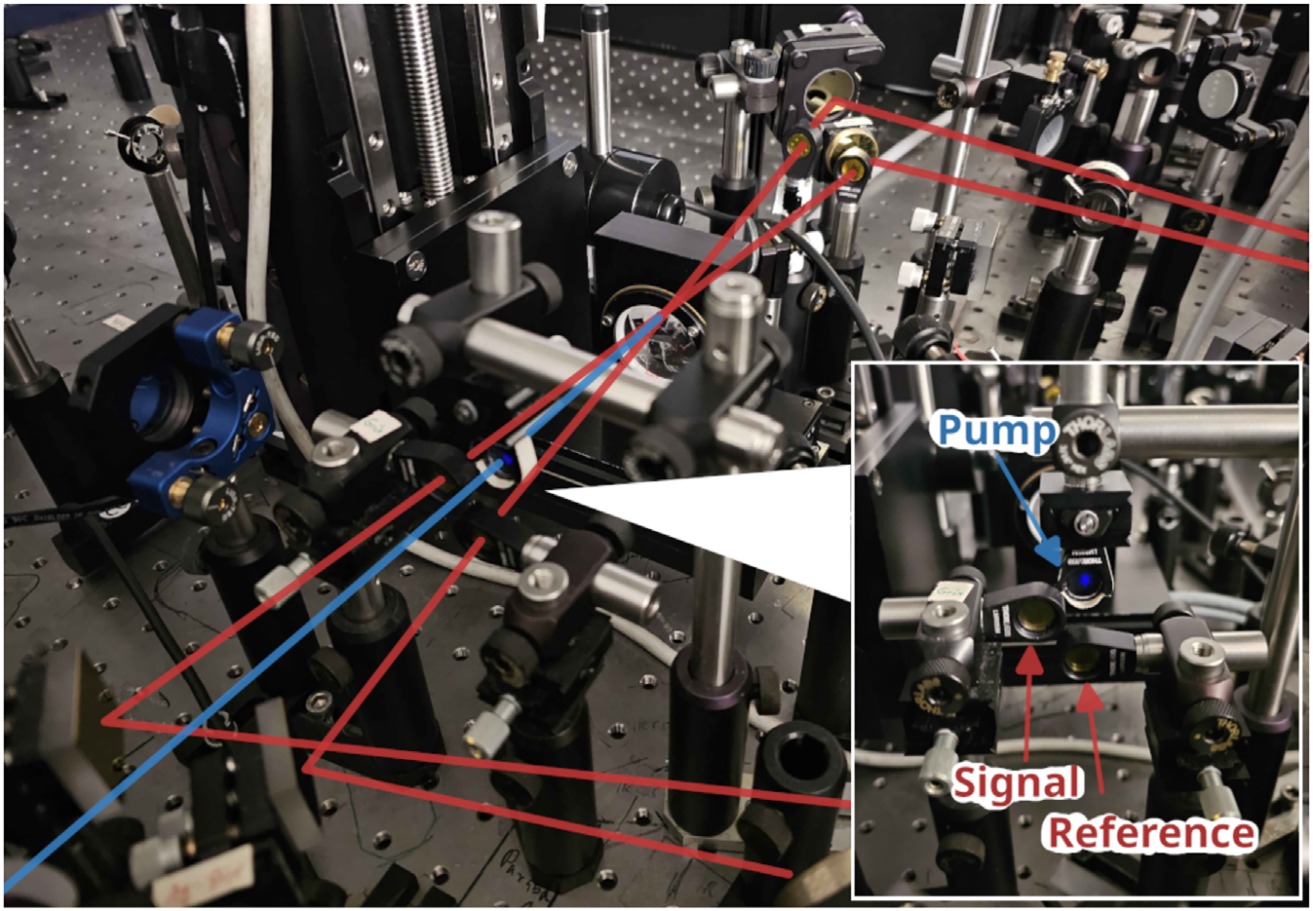
Referencing beam path, the signal and reference IR beams are focused into the sample by two f=100mm Zinc selenide lenses. After passing the sample, identical ZnSe lenses are used to collimate the beams before they are focussed into the spectrometer. The pump pulse is focused using a f=100mm calcium fluoride lens. After passing the sample, the pump pulse is directed to a power meter for characterizing of the excitation conditions.

This configuration effectively compensates for variations in optical path length and absorption arising from inhomogeneities in sample thickness and concentration, leading to an error with less than 1% noise. In practice, the referencing leads to an reduction of the standard deviation by a factor of two to five (Fig. 4). By implementing the presented referencing scheme in our system, the detectable change in absorbance is: 1000 shots: Δ*A*: 0.65 × 10^–3^; 10 000 shots: Δ*A*: 0.2 × 10^–3^; 20 000 shots: Δ*A*: 0.15 × 10^–3^; 30 000 shots: Δ*A*: 0.09 × 10^–3^.

**Fig. 4:**
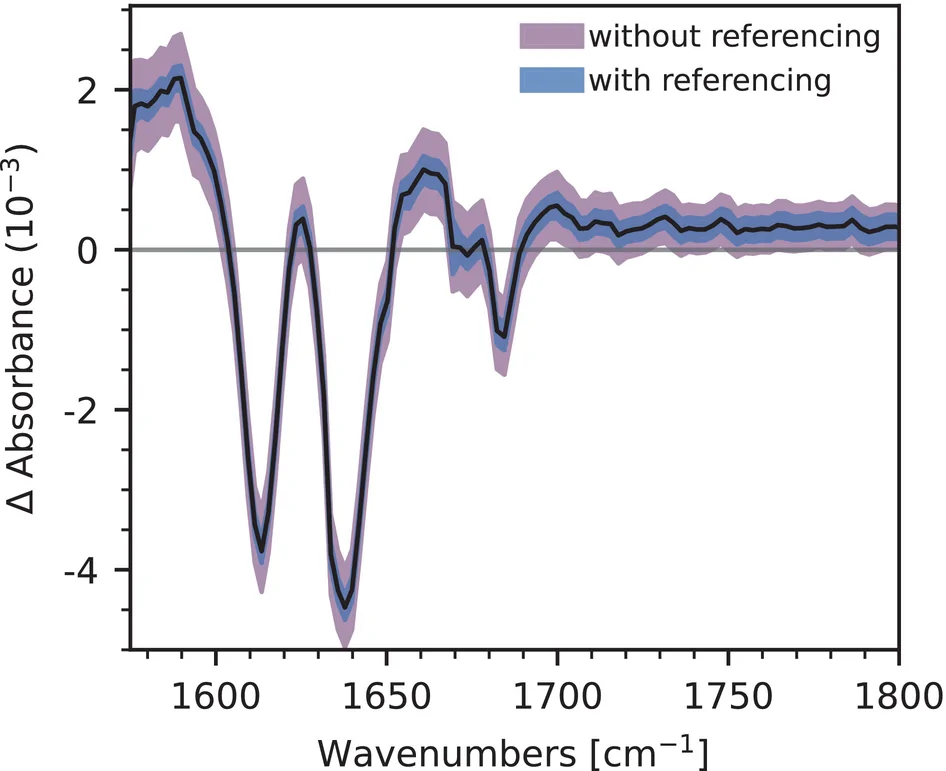
Error margings of transient absorption signals of Bacteriorhodopsin with referencing (blue) and without referencing (purple). Data are recorded with 600 shots over 85 scans. Referencing leads to well visible reduction of the standard deviation. This allows small signal contributions to to be distinguished from the background fluctuations as can be seen by the broad positive signal between 1700 and 1800 cm^−1^.

### Excitation conditions

For polarization resolved measurements, a careful characterization of excitation conditions is crucial to ensure photoselection. This includes analyzing the pulse energy, the spectrum and the spatial focus profile of the pump pulse. The pulse energy and spectrum of the pump pulse can be directly measured with a power meter and spectrometer.

#### Focus size

The spatial profile of the pump and probe beams are characterized using the knife‐edge method [9]. In this case, a razor blade is translated transversely across the beam during measurement of the transmitted power. The measured power P as function of the position $x_{k}$ is described by the integral of the beam profile. Assuming a gaussian beam profile, we obtain:

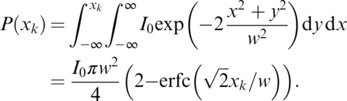

with $I_{0}$ being the maximal intensity, $\left(x^{2}+y^{2}\right)$ the radial component and w the beam‐waist. Fitting the error function to the measured data yields the beam waist w. However, there are several different definitions for the beam diameter. In our case, we use the $1/e^{2}$ and full width at half maximum, which are visualized in Fig. 5. Assuming a Gaussian beam, we obtain $d_{1/e^{2}}=2w$ and $d_{1/2}=2w\sqrt{2\log\left(2\right)}\;\approx \;0.59\;d_{1/e^{2}}$. In practice, however, the beam profile is rarely perfectly symmetrical. For asymmetrical beam profiles, the *x* and *y* components are determined separately using orthogonal knifes, yielding separate beam diameters d*x* and d*y*, as depicted in Fig. 5. This method can be easily implemented mounting orthogonal knifes on a sample cell, allowing simultaneous measurement of the profile of pump and probe beams at the pump–probe overlap.

**Fig. 5:**
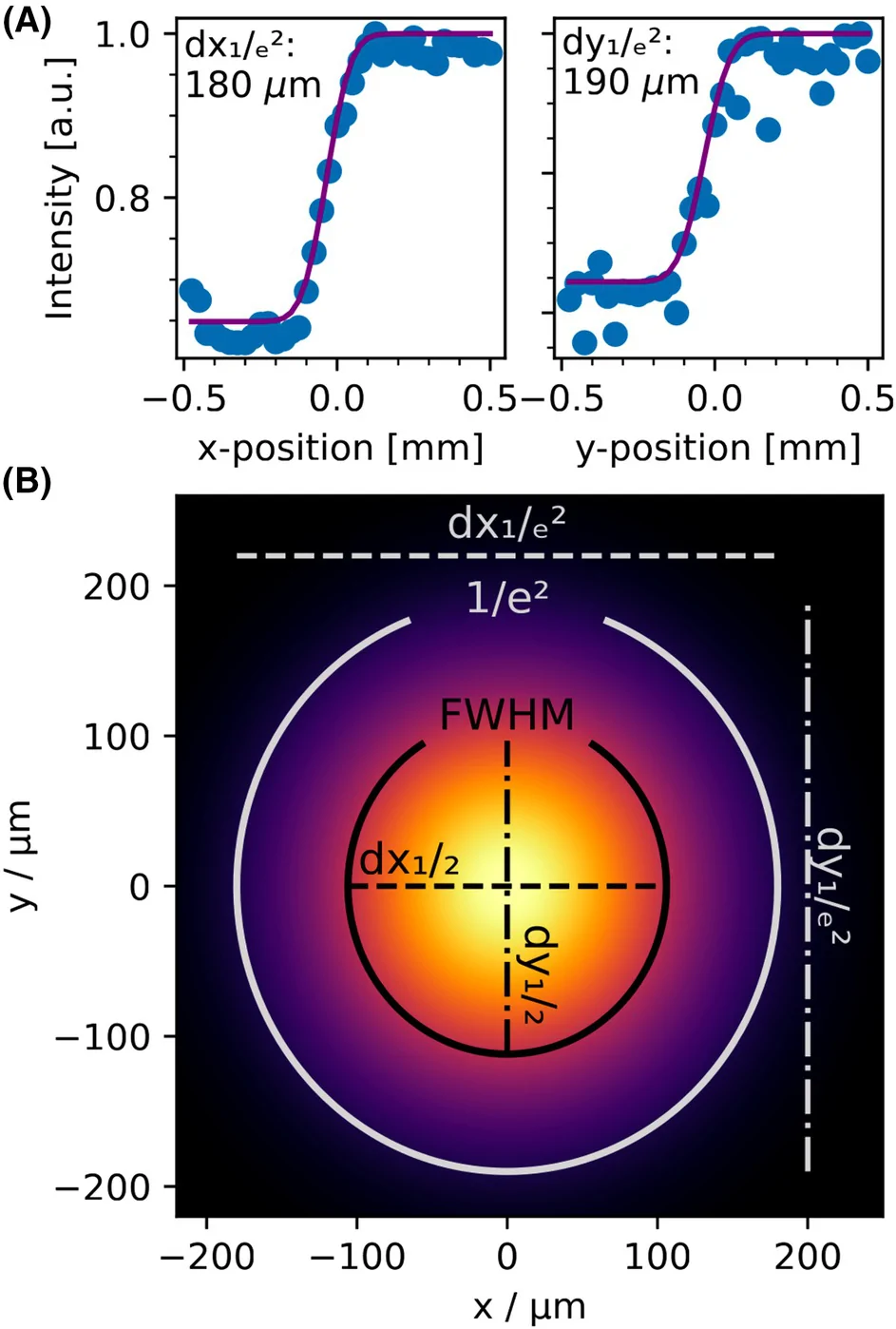
(A) Pump beam profile characterized using the knife‐edge method. (B) Color map simulating a gaussian beam with a beam diameter of $dx_{1/e^{2}}=180\;\mathrm{\mu m}$ and $dy_{1/e^{2}}=190\;\mathrm{\mu m}$. The focus profile is highlighted with contours at full width at half maximum (*d*_1/2_) and $1/e^{2}$ of the maximum value.

#### Time resolution and time zero position

The instrument response function of the setup is measured with a thin semiconductor wafer [10]. Depending on the pump wavelength, different types of semiconductors are more favorable. For a visible pump pulse, germanium (thickness 50 ± 10 μm) is typically a good choice.

When excited, the semiconductor exhibits a strong change in infrared absorption due to the generation of free charge carriers (electrons and holes). The resulting sharp step‐like increase in absorption reflects the cross‐correlation of the pump and probe pulses. By modeling the derivative of the signal with a Gaussian, we directly determine the time resolution (Fig. 6).

**Fig. 6:**
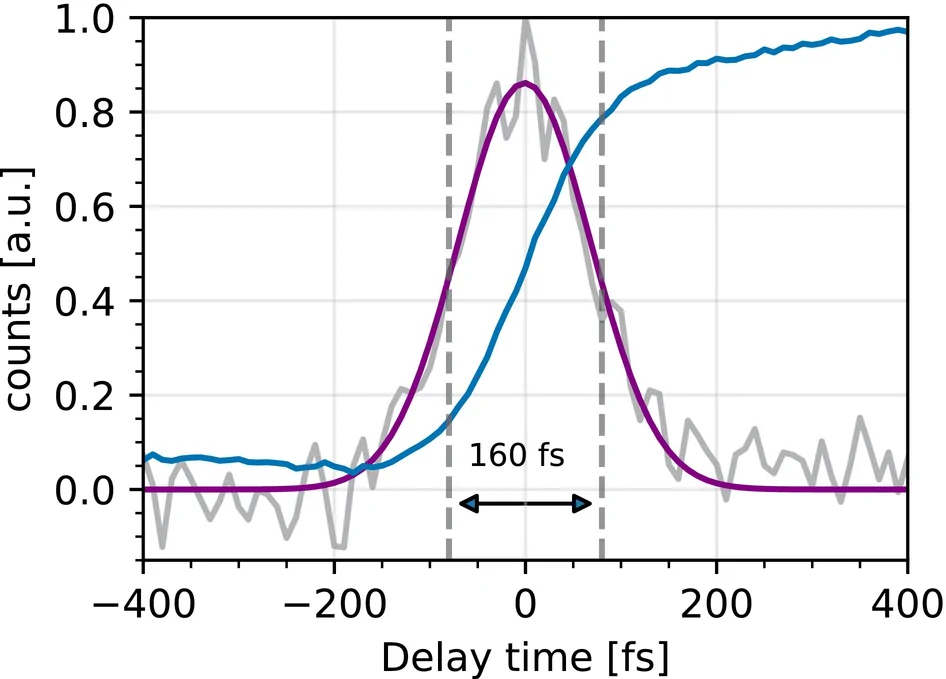
Averaged transient absorption signal measured in a germanium wafer (blue line) used to determine the instrument response function. The numerical derivative of the signal (gray line) represents the temporal cross‐correlation of the pump and probe pulses. A Gaussian fit (purple line) yields the full width at half maximum of the instrument response. In this example, the instrument response is 160 ± 10 fs.

### Polarization resolved IR spectroscopy

In polarization resolved Vis‐pump/IR‐probe experiments, photoselection creates an anisotropic subset of excited sample, oriented predominantly along the pump polarization. To achieve this, it is important to tune the excitation conditions, so that the pump pulse excites < 20% of the sample, as depicted in Fig. 7. At higher excitation rates, the photoselection and selective dipole orientation breaks down, leading to a more isotropic distribution of excited dipoles. This is typically controlled by attenuating the pump pulse and adjusting the pump focus.

**Fig. 7:**
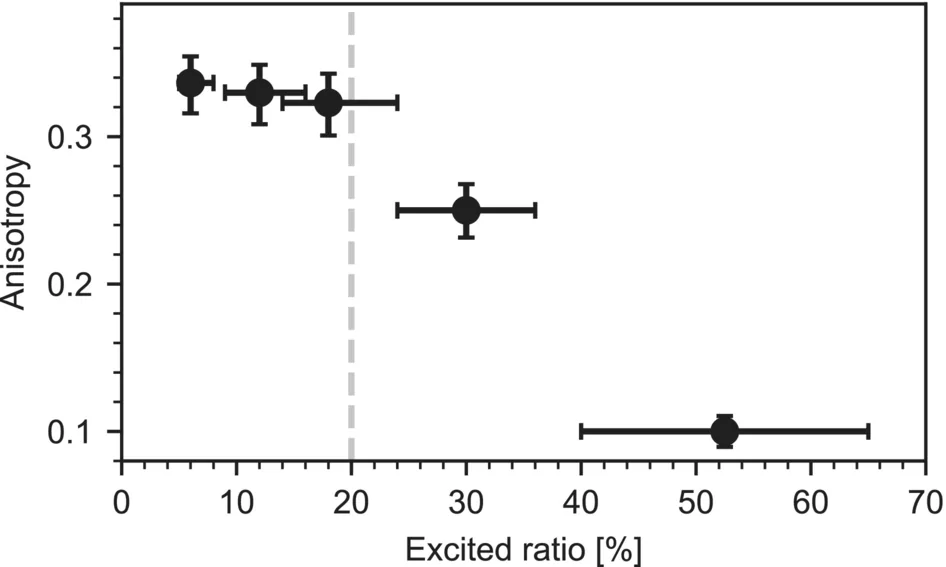
Anisotropy of the keto C=O band of chlorophyll a for different percentages of excited molecules. The power titration shows the loss of anisotropy upon increasing the percentage of excited molecules. We find that up to 20% (dashed gray line) the measured anisotropy is identical within the error margin (SD). Data adapted from a previous study [21].

#### Ratio of excited molecules

To estimate the ratio of excited molecules, we calculate the number of absorbed photons per molecule $N_{\mathrm{Ph}}/N_{M}$. The number of absorbed photons $N_{\mathrm{Ph}}$ per pulse is given by $N_{\mathrm{Ph}}=\frac{E_{p}\;\lambda }{h\;c}\left(1-10^{-\mathrm{OD}}\right)$. The number of molecules $N_{M}$ within the focal volume is determined by the beam waist w, sample thickness d and molecular concentration C. $N_{M}=\pi \;w^{2}\;d\;N_{A}\;C$, with *N* 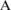 being the Avogadro constant.

#### Transient anisotropy

Alternating between perpendicular and parallel pump–probe configurations, we detect signal amplitudes for parallel $A_{∥}$ and perpendicular $A_{⊥}$ polarizations. The polarization dependence is described by the anisotropy r or dichroic ratio D, whereas dichroic ratio and anisotropy can be used interchangeably ($r=\left(D-1\right)/\left(D+2\right)$).

$$D=\frac{A_{P}}{A_{⊥}}\;\text{and}\;r=\frac{A_{P}-A_{⊥}}{A_{P}+2\;A_{⊥}}$$

The initial dichroic ratio or anisotropy value depends on the relative orientation between the probed vibrational tdm and the excited electronic tdm. Thus, we can calculate the relative angle θ between the probed vibrational tdm and the initially excited electronic tdm:

$$\theta =\arccos\left(\sqrt{\frac{5\;r+1}{3}}\right)$$

The dynamics of the anisotropy directly depend on the rotational dynamics of the system. For molecules in solution, rotational diffusion leads to anisotropy decay on a timescale of tens to hundreds of picoseconds. For rigid samples such as proteins, rotational diffusion is limited and vibrational anisotropy typically persist over longer timescales beyond the typically investigated time window of up to 1 ns.

### Data processing and data analysis

All data acquisition is performed using a custom‐developed Python software package by Till Stensitzki (https://github.com/Tillsten/messpy2d) [11]. The software is designed to control and synchronize all components of the pump–probe setup, including the delay stage, detector, polarization optics, sample holder, shutters and spectrograph. The software is modular and customizable, allowing integration of various hardware. It is open‐source and available on GitHub. Data analysis is also performed in Python, using the skultrafast package [11, 12]. The package supports a broad range of functionalities tailored to ultrafast spectroscopy, including time‐zero correction, global analysis and compartmental kinetic modeling. The software is actively maintained and accessible via GitHub [12]. In the following, we briefly touch the topic of data analysis, giving a short introduction on data modeling. An extensive in‐depth overview of analyzing time‐resolved absorption data can be found elsewhere [11, 13, 14, 15, 16, 17, 18, 19]. An illustrative example of data analysis is presented in Fig. 8.

**Fig. 8:**
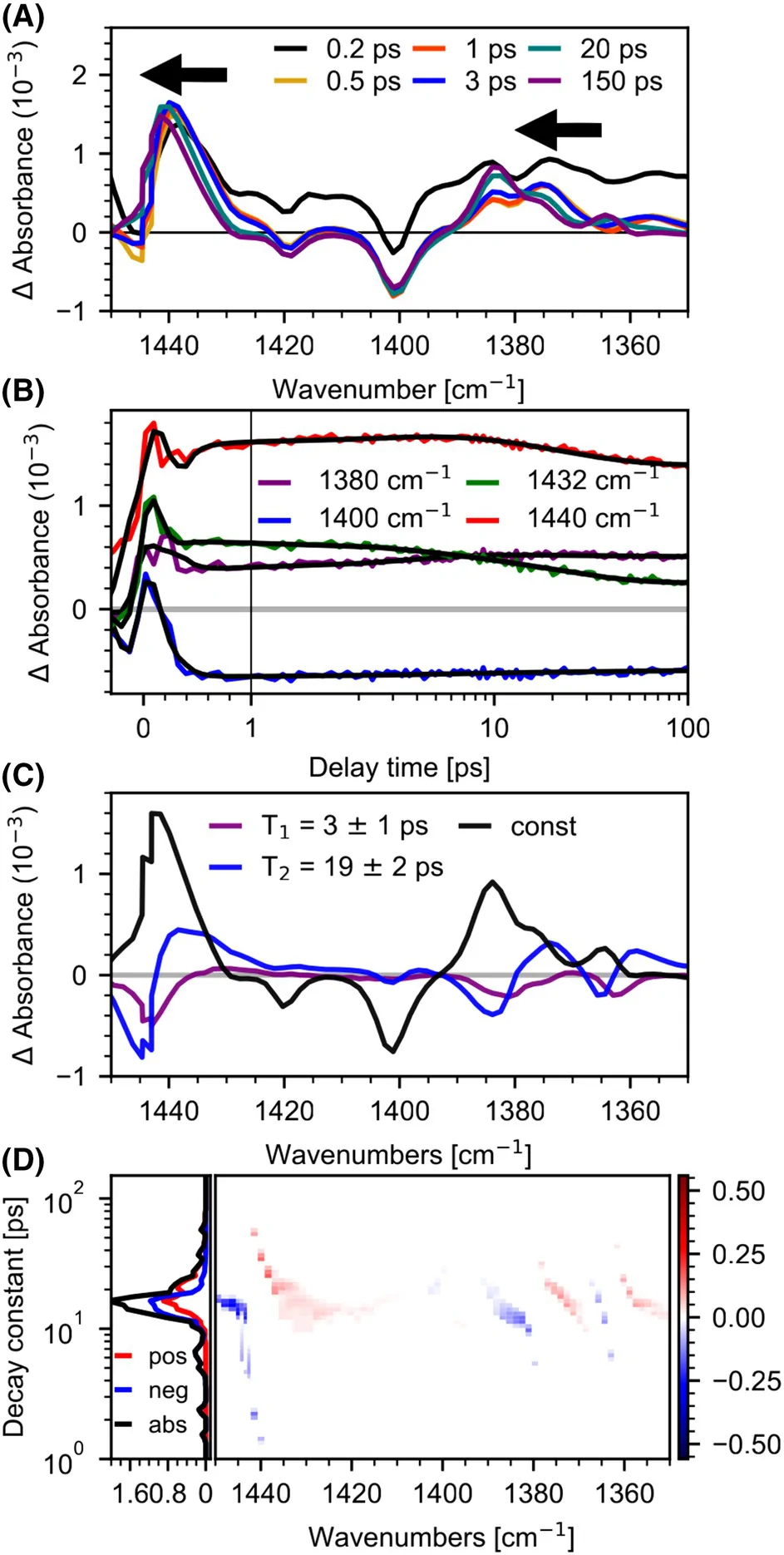
(A) Transient visible pump/infrared probe spectra of [Fe(2,6‐diquinolylpyridine)_2_]^2+^ at selected delay times. The positive peaks show a spectral blue shift (black arrows). (B) Transient absorption traces for selected wavenumbers. Black lines show modeling of the data, obtained from global analysis with an instrument response of 170 fs, two exponential decays and a constant term. (C) Decay associated spectra obtained from global analysis. Both decay associated spectra ($\tau_{1}$ and $\tau_{2}$) can be attributed to intramolecular energy redistribution and vibrational cooling. (D) Lifetime density map obtained with a set of 100 logarithmically distributed time points. At 10–20 ps, pairs of diagonal negative and positive contributions demonstrate a blue shift of signal as a result of energy redistribution and vibrational relaxation. Data adapted from a previous study [22].

#### Global analysis

The most common approach to analyzing time‐resolved spectroscopy data is to fit an exponential model. Assuming the process can be described with *n* sequential states, so that the exponential model is given by:

$$S\left(\nu ,t\right)=\sum_{i}^{n}A_{i}\left(\nu \right)\;\exp\left(\frac{-\left(t-t_{0}\right)}{\tau_{i}}\right)\;⊗\;\mathrm{IRF}$$

where $A_{i}\left(\nu \right)$ is the amplitude of the *n*‐th decay and $\tau_{i}$ the associated decay time. By fitting the dataset globally, the amplitudes $A_{i}\left(\nu \right)$ for each exponential decay give the decay associated spectra (Fig. 8C). Inspection of the decay associated spectra allows assignment of the spectral changes at different times to specific molecular processes, such as excited‐state decay, structural changes or charge and energy transfer. However, not all processes are well described by an sequential exponential model. Although global analysis works well for describing temporally well‐separated sequential processes, in more complex systems and for non‐exponential processes, such as energy and electron transfer or energy relaxation, a simple sequential model can not sufficiently describe the observed kinetics [16].

#### Lifetime analysis

A different approach to global analysis is lifetime density analysis. In lifetime density analysis, a sum of n (typically n>100) logarithmically distributed exponential functions with fixed decay times $\tau_{i}$ are fitted to the data. The resulting amplitudes $A_{i}$ for each exponential function yield a lifetime density map.

$$S\left(\nu ,t\right)=\sum_{i}^{n}A_{i}\left(\nu \right)\;\exp\left(\frac{-\left(t-t_{0}\right)}{\tau_{i}}\right)$$

Similar to global analysis, lifetime density analysis allows to identify exponential processes with distinct decay times, which are represented by horizontal contributions in the lifetime density map. In addition, non‐exponential processes exhibit broadened contributions across different decay times and spectral shifts appear as diagonal pairs of negative and positive contributions (Fig. 8D).

## Tips and tricks

### Vis/IR setup

- In this study, we employ a laser system with excellent shot‐to‐shot stability with a 128 × 128 element MCT‐array detector, for which the presented ratiometric referencing provides high signal stability. For less stable laser systems and different detector choices, ratiometric referencing may be less effective. In such cases, employing noise suppression algorithms [6] and edge‐pixel referencing [8] strategies can mitigate stability‐related noise.
- To reduce water absorption and ensure low humidity (< 5%), the laser table was enclosed and continuously flushed with dry air.

### Alignment

- Its useful to have an ‘alignment’ sample cell with orthogonal knifes, a 100 μm pinhole and semiconductor wafer(s) (typically one germanium and one indium arsenide wafer). This allows determination of the pump and probe foci, pump–probe overlap and time resolution at the exact sample position. The semiconductor signal is also used to set the time‐zero position of the delay stage.
- The ratio of excited molecules is calculated for the pump focus at full width at half maximum. This tends to overestimate the number of photons per molecule and gives an upper limit for the probability of excitation.

### Sample

- For proteins and other photo‐degrading samples, the sample decay over time leads to an increase in the ratio of excited molecules. This needs to be accounted for in polarization‐resolved experiments. The amount of photo‐degradation can be determined by comparing the visible absorption spectra before, during and after the experiment.

### Measurement settings

- As pump polarization is changed between scans, for photo‐sensitive biological samples one generally wants to set the number shot for each scan very low, which increases the total measurement time as a result of moving the mechanical delay. Balancing measurement time and shot count, we found that 500–1000 shots provides a good compromise for most samples.
- Selecting delay time points strongly depends on the investigated sample. Typically each scan starts with 10 pre‐zero points at −60 ps followed by a linear scan range around time zero and a logarithmic range for long delay times. For observing coherent oscillation choose a linear scan range for the duration of the oscillations. Linear time points are typically spaced by half of the instrument response. For the logarithmic range, from 1–5 ps up to 1 ns we typically choose 150 ± 50 time points.

## Discussion

Polarization‐resolved femtosecond Vis‐pump/IR‐probe spectroscopy is a very powerful method for investigating the structural dynamics with site‐ and structure‐specific sensitivity, allowing direct insight into its relation to function. Tracing the vibrational anisotropy provides additional orientational information by correlating the transition dipole of the electronic excitation with distinct vibrational modes.

To fully utilize the potential of Vis pump/IR probe spectroscopy for samples that are only available in limited quantities, we prepare samples custom‐built sample cells, requiring only approximately 50 μL of sample. However, although established referencing approaches can reach the detector noise floor [6, 8], they remain susceptible to noise arising from sample inhomogeneity and cell alignment. To further improve the S/N, we implemented a refined referencing, where the reference and signal beam are directed to pass through the exact same sample volume, reducing of the standard deviation by up to a factor of five.

Unfortunately, the spatial constraints in the current referencing scheme do not allow simultaneous detection of parallel and perpendicular signals. Thus, possible future improvement could employ mutual referencing between two probe beams of orthogonal polarization, where each polarization channel serves as the reference for the other, enabling concurrent acquisition of both polarizations.

In the following, we outline two studies of our group applying polarization‐resolved femtosecond Vis pump/IR probe spectroscopy:

### Phytochrome photoisomerization

A good example for applying polarization resolved femtosecond IR measurements are phytochromes. Phytochromes are light‐sensing proteins found in plants, fungi and bacteria. They regulate physiological responses to red (Pr‐state) and far‐red (Pfr‐state) light. The photoreaction is driven by a covalently bound bilin chromophore (Fig. 9, upper), which undergoes reversible photoisomerization to switch the protein between active and inactive states. After light excitation of the chromophore, the photoisomerization is achieved by the rotation of ring D around the methine bridge, accompanied by structural rearrangement in the protein manifold. In a recent study by our group, Yang *et al*. [20] investigated the ultrafast primary events of the photoreaction of bathyphytochrome Agp2 from *Agrobacterium fabrum*. A key part of the work comprises the femtosecond IR experiments, showing a broad continuum band (CB) (Fig. 9). Because averaging time was limited by photo‐degradation of the sample and limited sample availability, the presented referencing scheme was essential to achieve a sufficient S/N ratio to resolve the weak CB signal. Analyzing the polarization‐resolved measurements, it was possible to determine the relative angle between the CB and the electronic tdm.

**Fig. 9:**
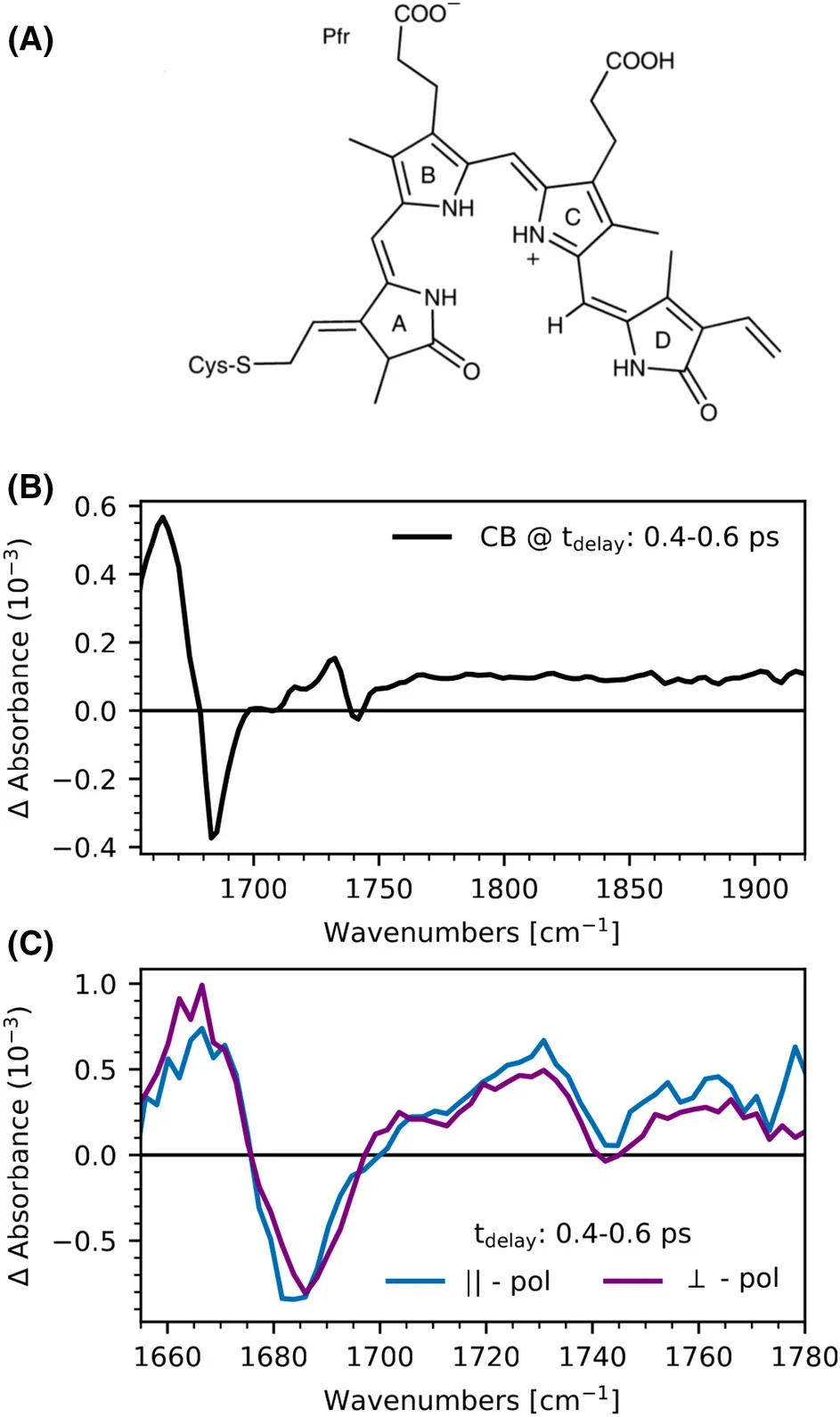
(A) Structure of the biliverdin chromophore of Agp2. Photoexcitation leads to the rotation of ring D around the methine bridge, converting the chromophore from the Pfr‐state to Pr‐state. (B) Isotropic infrared absorbance difference spectra of Agp2 in D2O (pD 8.0) averaged from 0.4 ps to 0.6 ps after excitation at 765 nm. The spectrum shows a broad continuum band across the whole spectral range. Prominent positive and negative peaks at 1666 cm^−1^ and 1685 cm^−1^ can be assigned to the carbonyl stretching vibration at ring D. (C) polarization resolved infrared absorbance difference spectra covering the low energetic part of the continuum band. The spectral range from 1750 cm^−1^ to 1780 cm^−1^ was analyzed for determining the relative angle of the continuum band. Data adapted from a previous study [20].

Complementing the experimental results with quantum mechanics/molecular mechanics calculations and molecular dynamics simulations, the broad CB can be assigned to transient proton‐loading of the water cluster around the chromophore. The full work is described in Yang *et al*. [20]

### Anisotropy excitation spectroscopy: Disentangling Q‐band absorption in chlorophyll *a*

Another possible application of polarization resolved femtosecond Vis pump/IR probe spectroscopy is anisotropy excitation spectroscopy [21]. In anisotropy excitation spectroscopy, we trace the anisotropy of a vibrational marker band as a function of excitation wavelength (Fig.10).

With this, we can correlate the change in anisotropy to altering contributions of the excited electronic state and disentangle spectral contributions of broad absorption spectra with overlapping absorption bands. A crucial requirement for this method is that the investigated sample has an IR band with high anisotropy contrast, meaning that the anisotropy varies significantly for different excitation wavelengths. In practice, it is convenient to work with the dichoric ratio instead of the anisotropy because the dichoric ratio is directly determined by modeling the experimental data. In the next step, the contribution of the two transitions, $S_{1}$ and $S_{2}$, is determined by modeling the dichroic ratio for different excitation wavelengths $D\left(\lambda \right)$ with a linear combination of the corresponding individual dichroic ratios $D_{S_{1}}$ and $D_{S_{2}}$. Assuming we can measure a pure dichroic ratio $D_{S_{1}}$ for the $S_{1}$ transition, the dependence of the dichroic ratio D(λ) is given by:

(1) $$D\left(\lambda \right)=(1-c\left(\lambda )\right)\;D_{S_{2}}+c\left(\lambda \right)\;D_{S_{1}}$$

where $c\left(\lambda \right)$is the relative contribution of the *S*_1_ transition between zero and one and $1-c\left(\lambda \right)$ is the relative contribution of the *S*_2_ transition. Applying this method to hexa‐coordinated chlorophyll *a*, we were able to separate *Q*_*x*_ and *Q*_*y*_ contributions to the Q‐band absorption quantitatively (Fig. 10), A detailed description of the method and results is provided in Zahn *et al*. [21].

**Fig. 10:**
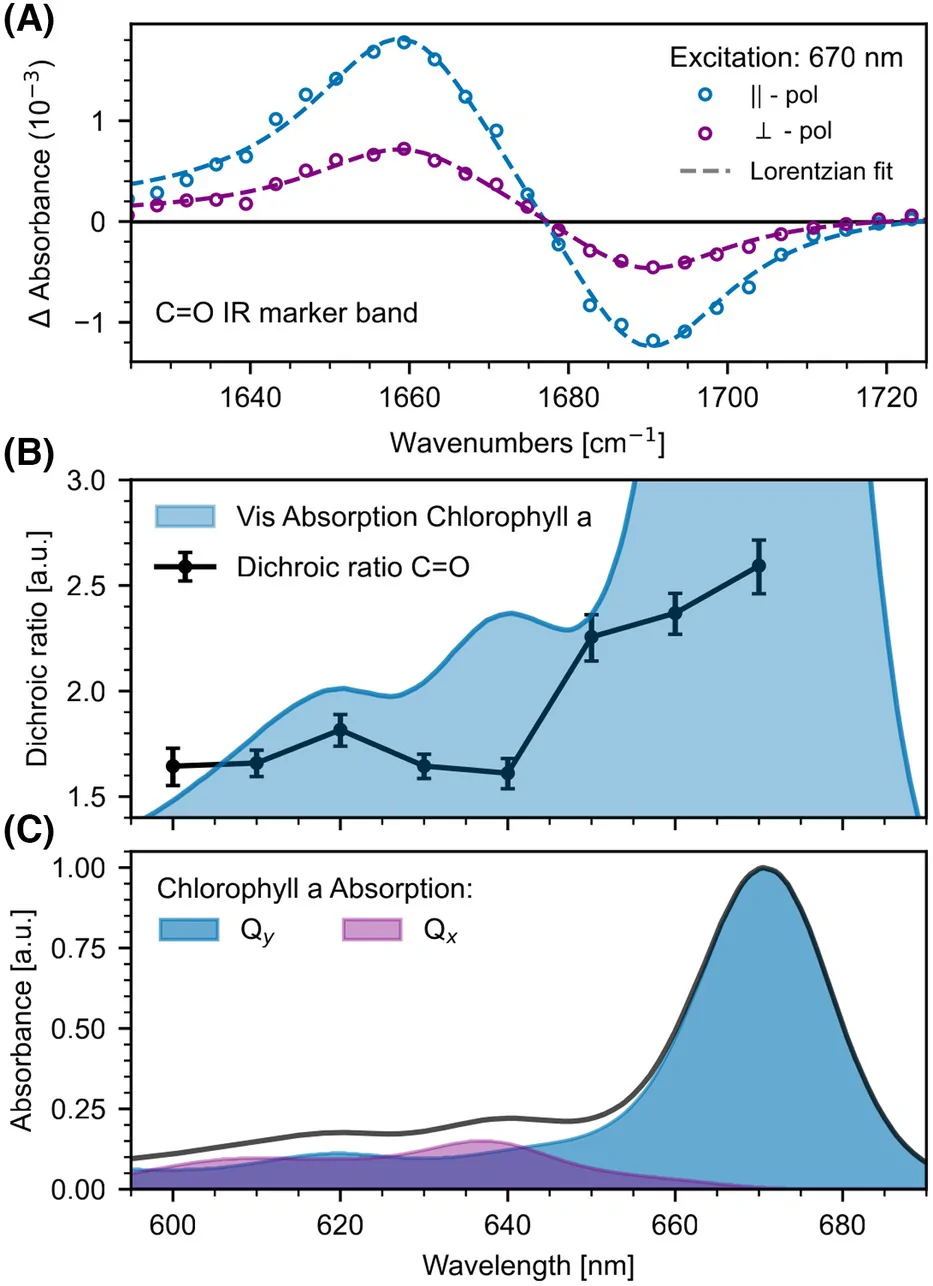
(A) Polarization resolved infrared absorbance difference spectra of hexa‐coordinated Chlorophyll a in pyrridine at a delay time of 10 ps after excitation at 670 nm. The bleaching (negative signal) and excited state absorption (positive signal) can be assigned to the keto C=O vibration. (B) Dichroic ratio of the keto C=O band upon excitation at different pump wavelengths (black), errors (SD, 1σ range). (C) Disentangling of *Q*_*x*_ and *Q*_*y*_ contributions to the Chl a absorption spectrum from modeling the change in dichroic ratio. Data adapted from a previous study [21].

## Conflicts of interest

The authors declare that they have no conflicts of interest.

## Author contributions

KH designed the experiment. CZ wrote the manuscript with input from KH.

## Data Availability

Datasets presented in the figures and the experimental setups are available upon request. Data adapted from previous studies [21,20] are available via the original publications.
